# Hydrogen-Rich Saline Inhibits NLRP3 Inflammasome Activation and Attenuates Experimental Acute Pancreatitis in Mice

**DOI:** 10.1155/2014/930894

**Published:** 2014-08-20

**Authors:** Jian-Dong Ren, Jie Ma, Jun Hou, Wen-Jin Xiao, Wei-Hua Jin, Juan Wu, Kai-Hua Fan

**Affiliations:** Department of Pharmacy, Chengdu Military General Hospital, No. 270, Rongdu Avenue, Jinniu District, Chengdu, Sichuan 610083, China

## Abstract

Increasing evidence has demonstrated that reactive oxygen species (ROS) induces oxidative stress and plays a crucial role in the pathogenesis of acute pancreatitis (AP). Hydrogen-rich saline (HRS), a well-known ROS scavenger, has been shown to possess therapeutic benefit on AP in many animal experiments. Recent findings have indicated that the NOD-like receptor family, pyrin domain-containing 3 (NLRP3) inflammasome, an intracellular multiprotein complex required for the maturation of interleukin- (IL-) 1*β*, may probably be a potential target of HRS in the treatment of AP. Therefore, in this study, we evaluated the activation of NLRP3 inflammasome and meanwhile assessed the degree of oxidative stress and inflammatory cascades, as well as the histological alterations in mice suffering from cerulein-induced AP after the treatment of HRS. The results showed that the activation of NLRP3 inflammasome in AP mice was substantially inhibited following the administration of HRS, which was paralleled with the decreased NF-*κ*B activity and cytokines production, attenuated oxidative stress and the amelioration of pancreatic tissue damage. In conclusion, our study has, for the first time, revealed that inhibition of the activation of NLRP3 inflammasome probably contributed to the therapeutic potential of HRS in AP.

## 1. Introduction

Acute pancreatitis (AP), a debilitating inflammation of the pancreas, is usually triggered by inappropriate intra-acinar activation of the trypsinogen. Although most cases of AP are mild or self-limiting, sometimes uncontrolled subsequent events including activation of immune cells and further systemic progression of inflammatory response are capable of converting AP to a severe form [1, 2]. Vigorous release of inflammatory mediators such as cytokines, chemokines, and reactive oxygen species (ROS) during systemic inflammatory responses results in a life-threatening condition with multiple-organ dysfunction, which carries substantial morbidity and mortality.

Currently, much research attention has been focused on the involvement of ROS and resultant oxidative stress in the inflammatory processes during the development of pancreatitis [3–5]. At the early stage of AP, several pathogenetic mechanisms, such as proteolytic damage, tissue ischaemia, and activation of polymorphonuclear leucocytes, stimulate the production of ROS that act as the molecular trigger of various inflammatory processes [6]. In general, the detrimental ROS can be detoxified either by endogenous antioxidants like glutathione (GSH) and superoxide dismutase (SOD) or when needed by exogenous dietary antioxidants like carotenoids, flavonoids, and vitamin E. However, once the production of ROS is excessive and overwhelms endogenous antioxidants, oxidative stress can occur with increasing cellular injury. Therefore, the theory that attack of oxidative stress is responsible for not only the amplification of pancreatic damage but also the progression of the disease from a local damage to a systemic organ failure is generally accepted as fact [3]. Therefore, supplementation with exogenous antioxidants is regarded as a beneficial therapeutic strategy, at least, a useful adjunctive option in the treatment of AP, as evidenced in some animal models [7–9].

Among numerous antioxidants, molecular hydrogen (H_2_) is emerging as a promising candidate in the therapeutic approaches of oxidative stress-implicated inflammatory diseases because this simple molecule is a selective scavenger of detrimental ROS, such as hydroxyl radical and peroxynitrite [10, 11]. In some animal experiments, hydrogen gas can be given by inhalation or more conveniently administered by injection when it dissolves in normal saline to achieve the saturation level (called hydrogen-rich saline, HRS). Recent studies, including our previous work, have provided evidence that treatment of HRS relieved the severity of pancreatitis in rats, describing the potential of HRS in the management of AP caused by different pathogenetic mechanisms [12, 13]. Although the molecular mechanism underlying the protective effect of HRS against AP has not been fully elucidated, it is well considered that the direct ROS elimination activity of HRS is mostly responsible for its therapeutic benefits due to the specific scavenging activity of cytotoxic ROS. Interestingly, emerging evidence has been presented that the potential of HRS to suppress the proinflammatory cascade also contributes to the reduction of the severity of the disease. Recently, the finding that activation of an intracellular multiprotein complex, the NOD-like receptor family, pyrin domain-containing 3 (NLRP3) inflammasome, is involved in the proinflammatory process in pancreas provides valuable insights into the pathogenesis of pancreatitis [14]. As the most well-characterized inflammasome, the NLRP3 inflammasome has been shown to possess the function of regulating the maturation and release of proinflammatory cytokine interleukin- (IL-) 1*β*. In previous work, we found that augmented secretion of IL-1*β* in rats suffering from trauma-induced pancreatitis was substantially depressed by the treatment of HRS, which implies a possibility that HRS is able to inhibit the activation of NLRP3 inflammasome, thereby contributing to the suppression of IL-1*β* production [13]. Thus, the herein study extended previous studies to evaluate the potential of HRS to inhibit NLRP3 inflammasome activation, which probably mediated the protective activity of HRS against experimental AP.

## 2. Materials and Methods 

### 2.1. Materials

Cerulein was purchased from Sigma (St. Louis, MO, USA). Antibodies against the p65 subunit of nuclear factor-*κ*B (NF-*κ*B p65), I-*κ*B*α*, phosphorylated-I-*κ*B*α* (p-I-*κ*B*α*), thioredoxin-interacting protein (TXNIP), NLRP3, p20 subunit of caspase-1 (casp-1-p20), *β*-actin, and horseradish peroxidase conjugated goat anti-rabbit antibody were obtained from Santa Cruz Biotechnology (Santa Cruz, CA, USA). Enzyme-linked immunosorbent assay (ELISA) kits for IL-1*β* and tumor necrosis factor-*α* (TNF-*α*) were obtained from R&D system (Minneapolis, MN, USA).

HRS was prepared as described previously [15]. In brief, H_2_ gas (0.4 MPa) was dissolved in normal saline (NS) for 6 hours to achieve a concentration of 0.6 mmol/L, and the concentration of H_2_ in NS was detected using a dissolved hydrogen analyzer (DH-35A; DKKTOA, Tokyo, Japan). The HRS was freshly prepared every week and then stored under atmospheric pressure at 4°C in an aluminum bag with no dead volume and sterilized by gamma radiation before use.

### 2.2. Experimental Mouse AP Model and Treatments

Male Balb/c mice (20–25 g) were obtained from the Experimental Animal Center of Sichuan University (Chengdu, Sichuan, China). Experimental AP model was induced by hourly intraperitoneal injections of cerulein (50 *μ*g/kg) to mice for 6 hours. One hour after the final injection, animals received an intraperitoneal injection of HRS at various doses (2, 4, and 8 mL/kg, every 20 min for 3 times) or control saline (air dissolved in NS at a pressure of 0.4 MPa for 6 h), respectively. Mice in sham group were treated with only NS. Tail vein blood samples were collected 2 h after the final administration of cerulein, and 24 h after the initiation of AP, all the animals were sacrificed by carbon dioxide asphyxiation followed by the collection of pancreatic tissue samples. All procedures involving the mice were carried out in accordance with the guidelines of the Institutional Animal Care and Use Committee of Sichuan University.

### 2.3. Analysis of NLRP3 Inflammasome Activation in Pancreas

Pancreas tissues were collected and homogenized in 20 mM phosphate buffer (pH 7.4) containing 0.5 mM butylated hydroxytoluene followed by a centrifugation at 4°C at 1,500 ×g for 15 minutes. Then cytoplasmic proteins in tissue homogenate were extracted using cytoplasmic extraction reagents according to the manufacturer's instructions (Pierce Biotechnology, Rockford, IL, USA). The BCA protein assay kit was employed to determine protein concentration. Subsequently, the extracted proteins were separated by SDS-polyacrylamide gels and then transferred to a polyvinylidene difluoride membrane (Millipore, Billerica, MA, USA). The membrane was incubated with blocking solution containing 5% skim milk for more than 1 h at room temperature and probed by 1 : 1,000 dilutions of rabbit polyclonal antibodies against TXNIP, NLRP3, and casp-1-p20, respectively. After washing, membrane was incubated with secondary goat anti-rabbit antibody conjugated to horseradish peroxidase for 1 h at room temperature. Immunoreactive proteins were visualized by chemiluminescence using the Western blotting detection system (Amersham Biosciences, Piscataway, NJ, USA).

### 2.4. Analysis of NF-*κ*B Activation in Pancreas

The nuclear and cytoplasmic proteins in pancreatic tissue homogenate were extracted using a protein extraction kit (Pierce Biotechnology, Rockford, IL, USA). The DNA-binding activity of NF-*κ*B p65 in nuclear extracts was detected using a sandwich ELISA kit (TransAM NF-*κ*B p65, Active Motif, Carlsbad, CA, USA). Briefly, oligonucleotide containing the NF-*κ*B consensus-binding site (5′-GGGACTTTCC-3′) was preimmobilized onto 96-well plates. The nuclear extract (1 *μ*g) diluted in complete lysis buffer was added to each well containing 30 *μ*L of complete binding buffer and incubated at room temperature for 1 h with mild agitation. After washing three times with wash buffer, the antibody against NF-*κ*B p65 subunit was added (1 : 1,000), followed by 1 h incubation at room temperature. After washing, the wells were incubated with the secondary antibody conjugated to horseradish peroxidase (1 : 1,000) for 1 h at room temperature. Then the DNA-binding activity of NF-*κ*B p65 was quantified by measuring the absorbance at 450 nm with a reference wavelength of 655 nm, after the wells were incubated with developing solution for 5 min at room temperature.

Moreover, the degradation of I-*κ*B caused by NF-*κ*B activation was evaluated by determining the phosphorylation of I-*κ*B*α* in pancreatic extracts using Western blotting assay as described above.

### 2.5. Analysis of SOD Activities in Pancreas

A colorimetric assay kit purchased from Cayman Chemical Company (Ann Arbor, MI, USA) was used to measure the activity of SOD in pancreas, which uses a tetrazolium salt for the detection of superoxide radicals generated by xanthine oxidase and hypoxanthine. Briefly, the pancreatic tissue was homogenized at 4°C in 20 mM HEPES buffer (containing 1 mM EGTA, 210 mM mannitol, and 70 mM sucrose, pH 7.2) and then centrifuged at 1,500 ×g for 5 min at 4°C. Then the radical detector (tetrazolium salt) and xanthine oxidase were added. After mixed, the sample was incubated at room temperature for 20 min and the absorbance was read at 450 nm. One unit (U) of SOD was defined as the amount of enzyme needed to produce 50% dismutation of superoxide radical. The total tissue protein concentration was determined by a commercial kit (Jiancheng Corp., Nanjing, China), and the activity of SOD was expressed as U/mg of protein.

### 2.6. Analysis of Malondialdehyde (MDA) and GSH Levels in Pancreas

Tissue MDA level, a marker of lipid peroxidation, was detected using a commercial MDA-586 assay kit (OxisResearch, Portland, OR, USA). In brief, pancreatic tissues were homogenized in 20 mM phosphate buffer (pH 7.4) containing 0.5 mM butylated hydroxytoluene followed by a centrifugation at 4°C at 1,500 ×g for 15 minutes. After protein concentration measurement, equal amounts of proteins were used in triplicate to react with a chromogenic reagent N-methyl-2-phenylindole to form a stable carbocyanine dye with a maximum absorption at 586 nm. The level of MDA in sample was then calculated with the standard curve obtained from the kit according to the manufacturer's instructions and expressed as *μ*mol/g of total protein.

The level of GSH in pancreas tissue was measured according to the method reported by Tietze [16]. In brief, pancreatic homogenate was centrifuged at 1,500 ×g for 20 min and 1 mL of the supernatant was mixed with 1 mL of 5% trichloroacetic acid for 30 min. Subsequently, 0.5 mL of Ellman's reagent (5,5-dithiobis-2-nitrobenzoic acid) and 3 mL of phosphate buffer (pH 8.0) were added. After mixed thoroughly, the absorbance was recorded at 412 nm. The GSH level in tissue was normalized against total protein (*μ*mol/g).

### 2.7. Pancreatic Enzymes Activity and Cytokines Concentrations in Plasma

Plasma samples were obtained from harvested blood specimens by centrifugation (1,800 ×g for 15 min at 4°C) and the amylase or lipase activity was analyzed through colorimetric method using a commercial kit for amylase or lipase (Jiancheng Co., Nanjing, China), respectively. The activity of amylase or lipase was expressed as units per liter (U/L). To determine the concentrations of proinflammatory cytokines in plasma, quantitative ELISA kits for IL-1*β* and TNF-*α* were used according to the manufacturer's instructions.

### 2.8. Histological Examination

Pancreatic tissues were fixed in formaldehyde and embedded in paraffin. Then samples were sectioned and stained with hematoxylin and eosin, using a standard staining procedure. Histopathological evaluation was performed under light microscope by an experienced laboratory pathologist who was blinded to the group identity for the samples. Edema, inflammatory infiltration, hemorrhage, and necrosis were evaluated in accordance with the scoring scale reported by Rongione et al. [17].

### 2.9. Statistical Comparisons and Presentation of Data

All experimental values are expressed as mean ± SE. Statistical comparisons among the groups were assessed by one-way analysis of variance (ANOVA) using SPSS 13.0 software. Differences in values were considered significant if *P* values < 0.05.

## 3. Results

### 3.1. HRS Inhibited NLRP3 Inflammasome Activation in Pancreas during AP

**Figure 1:**
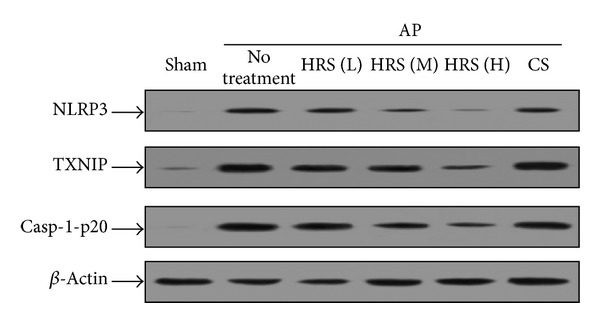
Evaluation of pancreatic NLRP3 inflammasome activation in mice by Western blot assay. The HRS at various doses or control saline (CS) was injected intraperitoneally to AP mice 1 h after the final injection of cerulein, respectively. Twenty-four hours later, animals in each group (*n* = 6) were killed and the pancreatic tissue samples were immediately collected and then homogenized on ice. After the measurement of protein concentration, the NLRP3 inflammasome activation was evaluated by detecting the protein levels of TXNIP, NLRP3, and casp-1-p20 in pancreatic homogenates. L: low dose (2 mL/kg × 3); M: middle dose (4 mL/kg × 3); H: high dose (8 mL/kg × 3).

The NLRP3 inflammasome is an intracellular multiprotein complex triggering inflammatory responses. The protein expression of NLRP3, a major component of this inflammasome, was remarkably increased in the progression of AP. Moreover, the development of cerulein-induced pancreatitis conspicuously stimulated the expression of TXNIP, the critical regulator of NLRP3 activity. Similar elevation was also found in the protein expression of p20 subunit of activated caspase-1, used as one of the markers for the NLRP3 inflammasome activation. However, treatment of HRS dose-dependently diminished expression levels of NLRP3, TXNIP, and casp-1-p20, exhibiting the significant inhibitory activity on the NLRP3 inflammasome activation (Figure 1).

### 3.2. HRS Inhibited NF-*κ*B Activation in Pancreas during AP

**Figure 2:**
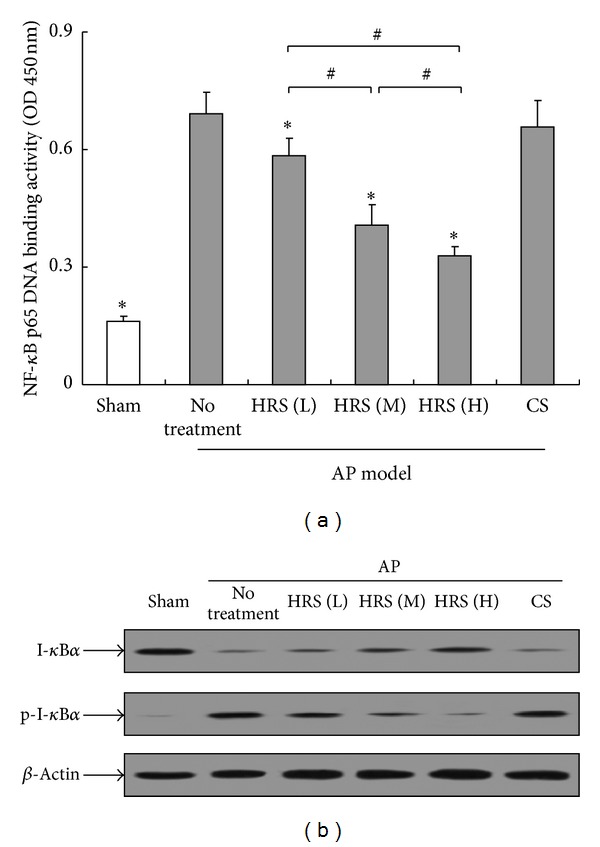
Evaluation of pancreatic NF-*κ*B activation in mice. The HRS at various doses or control saline (CS) was injected intraperitoneally to AP mice 1 h after the final injection of cerulein, respectively. The pancreatic tissue samples were collected from sacrificed animals in each group (*n* = 6) 24 h after the initiation of AP and then homogenized on ice. After the measurement of protein concentration, NF-*κ*B activation was evaluated by measuring the DNA-binding activity of NF-*κ*B p65 in nuclear extracts using an ELISA kit (a) and by detecting the protein levels of I-*κ*B*α* and p-I-*κ*B*α* in pancreatic homogenates (b). L: low dose (2 mL/kg × 3); M: middle dose (4 mL/kg × 3); H: high dose (8 mL/kg × 3).

It was known that NF-*κ*B is ubiquitous transcription factor that controls proinflammatory gene expression during the development of inflammation related diseases including AP. In present study, the DNA-binding activity of NF-*κ*B p65 in pancreas measured by the ELISA increased significantly in response to the induction of pancreatitis, indicating the activation of NF-*κ*B, whereas it was substantially depressed by the administration of HRS in a dose-response manner (Figure 2(a)). In line with the ELISA data, protein expression of cytoplasmic p-I-*κ*B*α* in pancreatic homogenates from AP mice rose markedly, demonstrating the degradation of I-*κ*B. Likewise, HRS showed a dose-dependent inhibition on the degradation of I-*κ*B, which was in agreement with the inhibitory effect on NLRP3 activity (Figure 2(b)).

### 3.3. HRS Ameliorated Pancreatic Damage in AP Mice

**Figure 3:**
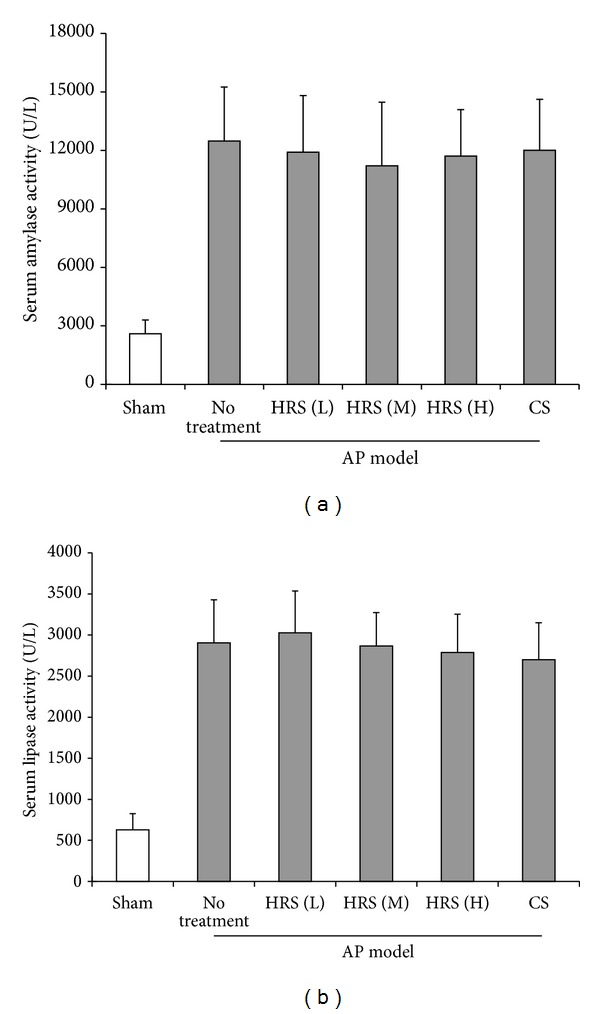
Determination of plasma amylase and lipase activities in mice. Experimental AP model was induced by hourly intraperitoneal injections of cerulein (50 *μ*g/kg × 6) to mice. Then the HRS at various doses or control saline (CS) was injected intraperitoneally to AP mice 1 h after the final injection of cerulein, respectively. Animals in each group (*n* = 6) were sacrificed by CO_2_ asphyxiation 1 h after the final administration of HRS, and immediately the blood and tissue samples were collected. The amylase and lipase activities in plasma were determined with a colorimetric method using the commercial kits for amylase and lipase, respectively. L: low dose (2 mL/kg × 3); M: middle dose (4 mL/kg × 3); H: high dose (8 mL/kg × 3).

**Figure 4:**
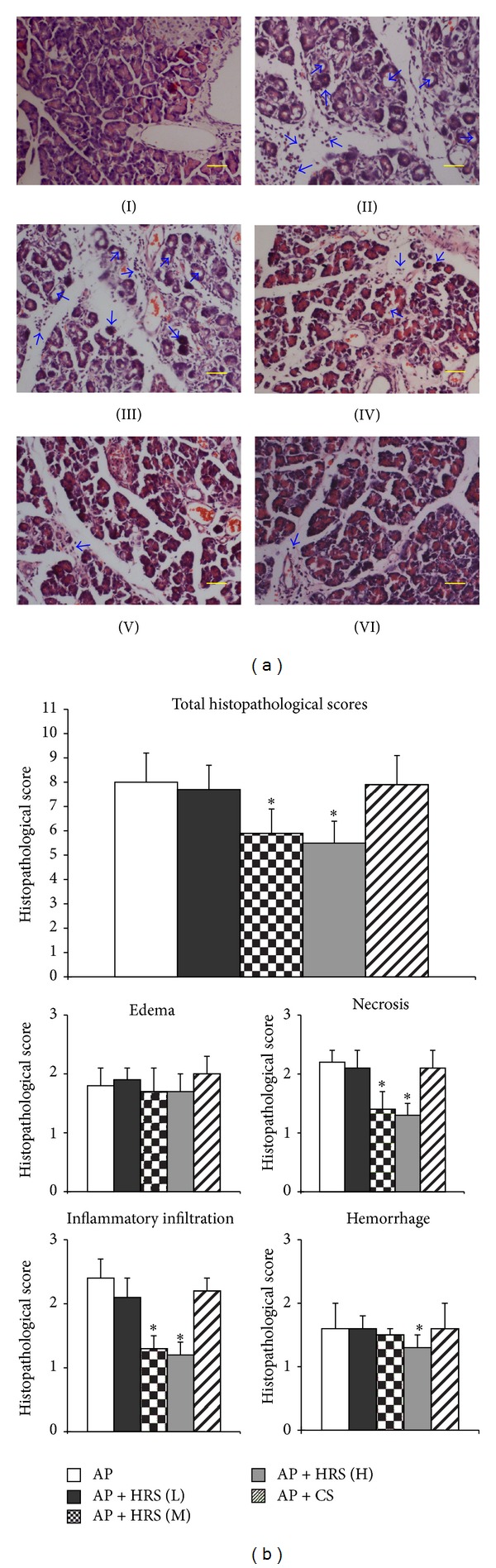
Histopathological examination of pancreatic tissue from mice. The HRS at various doses or control saline (CS) was injected intraperitoneally to AP mice 1 h after the final injection of cerulein, respectively. One hour later, animals in each group (*n* = 6) were killed and the pancreatic tissue samples were immediately collected. After fixed in formaldehyde and embedded in paraffin, the samples were sectioned and stained with hematoxylin and eosin, using a standard staining procedure. Histopathological evaluation was performed under light microscope and the scores were calculated according to histopathological changes including edema, inflammatory infiltration, hemorrhage, and necrosis. (a) Images of histological sections; I: sham; II: AP model group; III: AP + CS; IV: AP + HRS (L); V: AP + HRS (M); VI: AP + HRS (H); scale bars = 50 *μ*m. Significant histopathological changes like cytoplasmic vacuole generation, inflammatory cells infiltration, and parenchyma necrosis were indicated by blue arrows. (b) Histopathological scores. *P < 0.05, compared with AP group.

As shown in Figures 3 and 4, experimental AP was induced by cerulein administration in mice with significant elevated plasma pancreatic enzymes activities and pathological damage in pancreas. There were no significant changes of plasma amylase and lipase activities in AP mice after the treatment of HRS at various doses (Figure 3). Moreover, only at the higher doses (4 and 8 mL/kg × 3), HRS conferred favorable histopathological changes in pancreatic tissues, mainly characterized by reduced cytoplasmic vacuole generation, alleviated inflammatory infiltration, and parenchyma necrosis, which was paralleled with lower histopathological scores (Figures 4(a) and 4(b)).

### 3.4. HRS Decreased Plasma Cytokines Concentration in AP Mice

**Figure 5:**
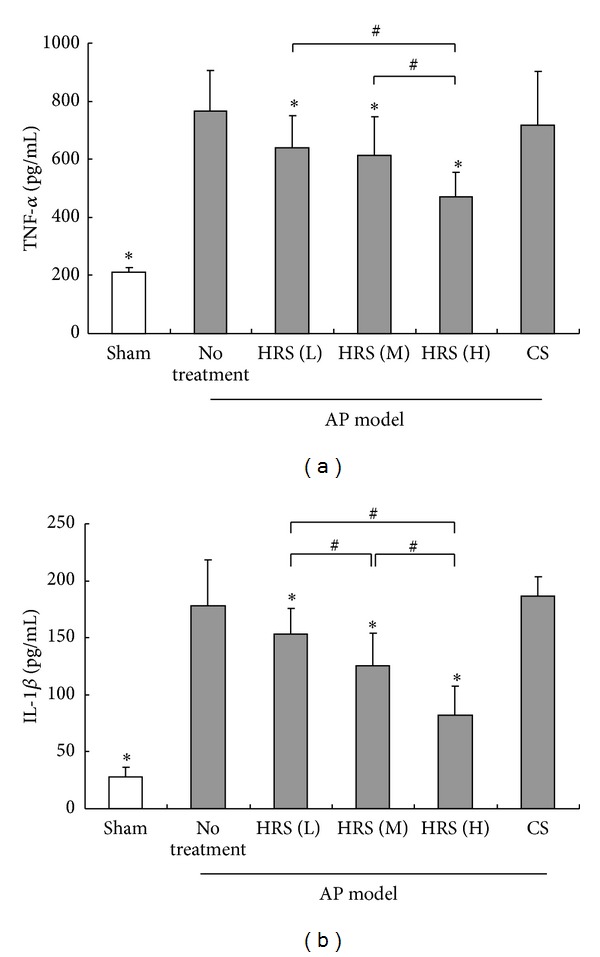
Measurement of plasma cytokines concentration in mice. The HRS at various doses or control saline (CS) was injected intraperitoneally to AP mice 1 h after the final injection of cerulein, respectively. One hour later, animals in each group (*n* = 6) were killed and the blood samples were immediately collected. The plasma concentrations of TNF-*α* and IL-1*β* were measured using quantitative ELISA kits according to the manufacturer's instructions. *P < 0.05, compared with the group of AP mice with no treatment; ^#^ *P* < 0.05, compared in groups of AP mice treated with HRS at different doses; L: low dose (2 mL/kg × 3); M: middle dose (4 mL/kg × 3); H: high dose (8 mL/kg × 3).

The proinflammatory cytokines TNF-*α* and IL-1*β* are known to mediate amplification of inflammatory process during pancreatitis. Here it was shown that plasma concentrations of TNF-*α* and IL-1*β* markedly increased in mice suffering from cerulein-induced AP, which were substantially abolished by the treatment of HRS (Figure 5). In addition, a significant dose-dependent effect of HRS to decline the plasma IL-1*β* level was observed; however, middle dose of HRS (4 mL/kg × 3) failed to demonstrate superiority in reducing TNF-*α* release in AP mice when compared with the low dose (2 mL/kg × 3).

### 3.5. HRS Inhibited Oxidative Stress in Pancreas during AP

**Figure 6:**
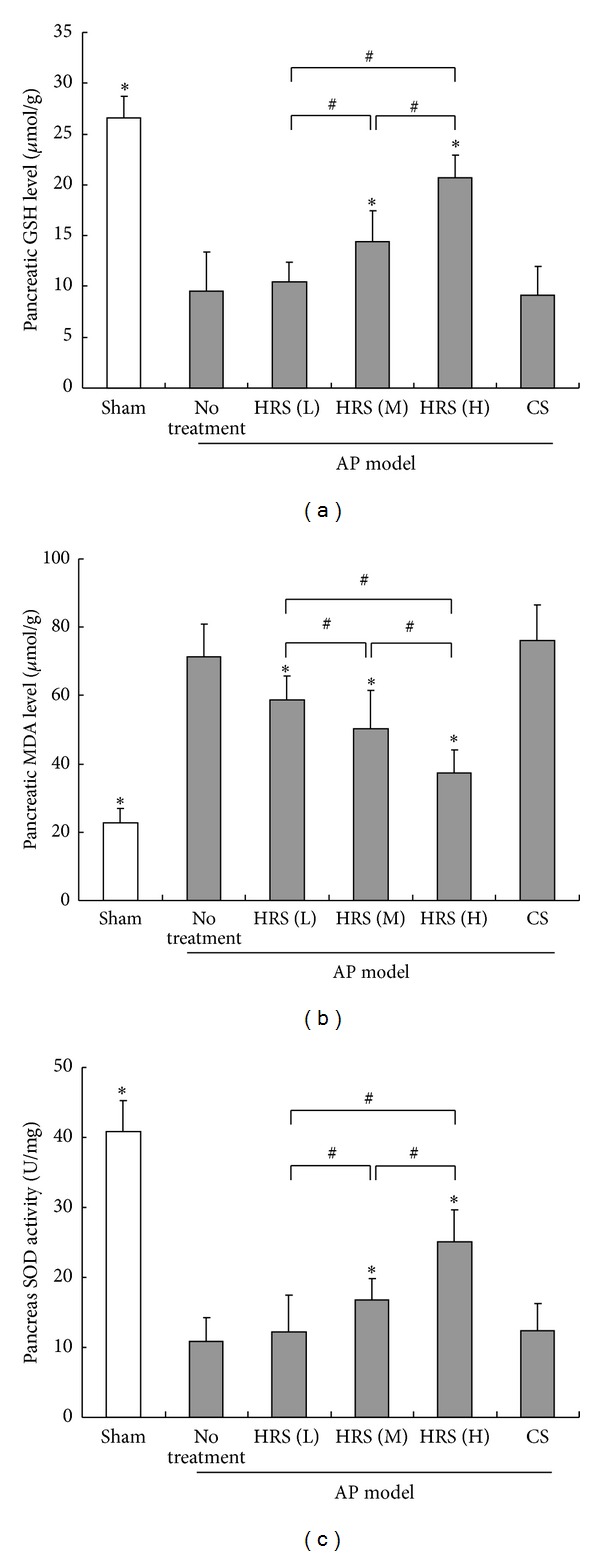
Determination of pancreatic tissue levels of GSH, MDA, and SOD in mice. The HRS at various doses or control saline (CS) was injected intraperitoneally to AP mice 1 h after the final injection of cerulein, respectively. One hour later, animals in each group (*n* = 6) were killed and the pancreatic tissue samples were immediately collected and then homogenized on ice. After the measurement of protein concentration, the levels of GSH (a) and MDA (b) and the activity of SOD (c) in pancreatic homogenates were determined according to the methods reported previously. *P < 0.05, compared with the group of AP mice with no treatment; ^#^ *P* < 0.05, compared in groups of AP mice treated with HRS at different doses; L: low dose (2 mL/kg × 3); M: middle dose (4 mL/kg × 3); H: high dose (8 mL/kg × 3).

In order to verify the potential of HRS to reduce oxidative stress during AP, we measured the changes of pancreatic MDA level, an indicator of lipid peroxidation in oxidative stress process, as well as the endogenous antioxidants GSH and SOD. As expected, enhanced oxidative stress in pancreas was observed in mice after the initiation of AP, which was characterized by increased amount of MDA and decreased SOD activity and GSH level. In contrast, administration of HRS dose-dependently diminished the MDA production, accompanied with the elevated levels of SOD and GSH (Figure 6). Moreover, the capacity of HRS to help recruit endogenous antioxidants was observed only at the middle (4 mL/kg × 3) or high dose (8 mL/kg × 3) in our experiment.

## 4. Discussion

Over the past years, there has been continuing controversy about the benefits of supplementation with exogenous antioxidants for the management of oxidative stress related inflammatory diseases largely because once it was considered that these antioxidants only served as ROS scavengers and thereby helped correcting the pro- or antioxidative imbalance [18]. However, recent findings that elimination of ROS by antioxidants suppressed activation of NLRP3 inflammasome and the resultant maturation of IL-1*β* have shed new light on understanding the roles of antioxidants in pathogenesis of inflammation related diseases [19]. Here we found that, although HRS failed to suppress plasma pancreatic enzymes activity, it significantly attenuated the pancreatic damage in mice in response to the induction of pancreatitis, together with the impaired NLRP3 inflammasome activity, suggesting that the potential of HRS to block the NLRP3 inflammasome activation was probably involved in the amelioration of pancreatic injury. Moreover, the data from this study have demonstrated the possible mechanism by which HRS inhibited the activation of NLRP3 inflammasome. Firstly, because the NF-*κ*B activation is shown previously to be the traditional priming signal to induce the NLRP3 expression, blockade of NF-*κ*B activity by HRS, with the following downregulation of expression of NLRP3, probably conferred the suppression of NLRP3 inflammasome activation. Furthermore, treatment of HRS promoted the elimination of ROS and thereby reduced the expression of TXNIP, the ROS-sensing protein required for NLRP3 activation, also resulting in the blockade of NLRP3 inflammasome activation.

As an important component of innate immune system, the NLRP3 inflammasome was initially found to be activated by pathogens [20–23]. In recent years, some endogenous molecules known as damage-associated molecular patterns (DAMPs), such as extracellular glucose, uric acid, amyloid-*β*, and hyaluronan, have been demonstrated to activate NLRP3 inflammasome, playing important roles in the metabolic disorders and sterile inflammatory responses including type II diabetes, gout, Alzheimer's disease, and tissue injury after trauma [24–27]. As for AP, some known extracellular NLRP3 activators like DNA, adenosine triphosphate (ATP), high mobility group box 1 (HMGB1), and nucleotide binding oligomerization domain 2 (NOD2) are all associated with the inflammation during the development of pancreatitis. Moreover, some researches, including our previous work, have revealed that heparan sulfate (HS), a degradation product of extracellular matrix known as an important member of DAMPs, triggered intracellular proinflammatory responses, resulting in the aggravation of pancreatitis [28–30]. Therefore, the above-mentioned findings hint at a possible fact that HS might act as a previously unknown agonist of NLRP3 inflammasome and thereby promote the progression of AP. In this context, there arises an interesting question required to be addressed by future work, that is, whether HRS possesses the ability to inhibit the presumed HS-induced activation of NLRP3 during the development of pancreatitis. Obviously, the elucidation of this question is of considerable significance to further understand the beneficial roles of hydrogen molecule in AP.

In addition, although the mechanism underlying the therapeutic potential of HRS is probably shared by other antioxidants due to their ROS scavenging capacity, HRS still exhibits significant advantages in the treatment of oxidative stress-implicated diseases. At first, it was known that mammalian species are lacking endogenous detoxification systems for the hydroxyl radical, which is considered as one of the strongest oxidant species having harmful reactions to nucleic acids, lipids, and proteins, further causing irreversible cellular damage. Compared with other known exogenous antioxidants, hydrogen molecule can selectively quench hydroxyl radical forming water due to the unique molecular structure and chemical property, meanwhile without excessively interfering the physiological roles of the beneficial ROS like superoxide anions [10]. Moreover, HRS can be easily injected or administered orally and even used as the dissolvent for other therapeutic agents. Therefore, the advantages of HRS mentioned above make it a superior candidate for the detoxification of oxidative stress in clinical application.

In summary, our work has, for the first time, revealed that HRS can act as an antagonist of NLRP3 inflammasome resulting in attenuation of cellular inflammatory processes and consequently relieve the pancreatic injury during AP. This finding allows for a further understanding of the mechanism underlying the protective effects of HRS against AP and furthermore marks the NLRP3 inflammasome as a potential target for future therapeutic approaches of this dangerous disease.
